# Topology of chromosome centromeres in human sperm nuclei with high levels of DNA damage

**DOI:** 10.1038/srep31614

**Published:** 2016-08-25

**Authors:** Ewa Wiland, Monika Fraczek, Marta Olszewska, Maciej Kurpisz

**Affiliations:** 1 Institute of Human Genetics, Polish Academy of Sciences, Strzeszyńska 32, 60-479 Poznań, Poland

## Abstract

Several studies have shown that the ‘poor’ sperm DNA quality appears to be an important factor affecting male reproductive ability. In the case of sperm cells from males with the correct somatic karyotype but with deficient spermatogenesis, resulting in a high degree of sperm DNA fragmentation, we observed changes in the preferential topology of the chromosome 7, 9, 15, 18, X and Y centromeres. The changes occurred in radial localization and may have been directly linked to the sperm chromatin damage. This conclusion is mainly based on a comparison of FISH signals that were observed simultaneously in the TUNEL-positive and TUNEL-negative sperm cells. The analyzed cells originated from the same ejaculated sample and FISH was performed on the same slides, after *in situ* TUNEL reaction. Based on the observed changes and previous data, it appears that the sperm nucleus architecture can be disrupted by a variety of factors and has a negative influence on spermatogenesis at the same time. Often, these factors coexist (e.g. chromosomal translocations, aneuploidies, a higher DNA fragmentation, abnormal seminology), but no direct correlations between the factors were observed.

Chromatin is packed in a specific way into the 23 chromosomes inside human spermatozoa. The differences in the chromatin organization within sperm and somatic cells chromosomes are due to differences in the molecular structure of the protamine DNA-complexes in spermatozoa. Chromatin is converted during spermiogenesis. First, somatic histones are gradually replaced by transition proteins (TP). Next, the transition proteins of elongated spermatids are replaced by protamines. When protamines bind to DNA, they cause the DNA particles to roll up into concentric circles, consequently creating a structure involving strong condensate toroids. Such toroids comprising approx. 50 kpz DNA and sperm DNA exhibit the most tightly compacted form of eukaryotic DNA^1^,^2^. The exchange of histones for the proteins found in sperm cells toroids is linked to the fact that in spermatids, DNA must undergo a series of programmed breaks and subsequent repairs. This process is believed to necessitate topoisomerase II activity, and these DNA breaks appear to correspond to the presence of DSB (double strand breaks)^1^,^3^. At later stages of spermiogenesis, DNA breaks are not detectable and therefore appear to be of a transient, indicating the presence of a DNA repair mechanism^4^. In a mature spermatozoa, approximately 5–15% of the DNA remains bound to histones variants^5^. At the sites where histones occur, the nucleosome structure is preserved^6^. It has been demonstrated that the telomeric regions of spermatozoa chromosomes have a nucleosomal structure^7^. It is also known that both histones and protamines are associated with the centromeric sequences of sperm chromosomes^8^. Histones have been found in spermatozoa DNA domains that are susceptible to exogenous and endogenous nucleases^9^,^10^. It has been suggested that the nucleosomes in the sperm cell chromosomes might be associated with the genes that undergo transcription during the early stages of zygotic development^5^,^11^. Alternatively, it might be assumed that sperm cell histones are associated with all specific coding sequences^5^,^10^. It is very likely that sperm chromatin can contribute to the transmission of information to the zygote and that theis transmission might be crucial for paternal pronuclear chromatin remodeling^12^.

The structural layout of the sperm DNA is presented as the so-called donut-loop model^13^. In turn, a model of the larger structural elements of sperm cell chromosomes (>300 nm) was proposed by Mudrak *et al.*^2^. In this model, chromosomes are constructed from chromatin fibres that are approximately 1,000 nm in diameter. Thisw 1,000-nm fibre comprises two connected globular structures (2 × 500 nm), which together form a groove along the long axis of the fibre. Each of these globular structures is thought to comprise 100–200 toroids, which could correspond to 3–5 Mpz DNA^2^. It is assumed that sperm cell chromosomes occupy individual territories and that, like their chromatin, they are 4–6 times more tightly condensed than the chromosomes of the somatic cells^1^,^2^. Studies suggest that sperm chromosome centromeres form an inner cluster (or 2–3 smaller clusters) in the area of the nucleus termed the chromocentre^14^,^15^. Unlike the centromeres, chromosome telomeres are located at the nuclear periphery of the spermatozoa nucleus and form dimers and tetramers. Dimers are created through of the interaction of the telomeres of the short (p) and long (q) arms of the same chromosome; thus, chromosomes form a hairpin-shaped loop conformation^16^.

It has been suggested that the described intranuclear architecture is of considerable importance for the correct decondensation of chromatin in the male pronucleus^16^,^17^. It appears that the chronology of the zygotic genome activation is more dependent on the changes in the chromatin structure than on transcription factor activation^18^,^19^. It must be emphasized, however, that the relationship between the chromatin organization and the sperm cell function remains under investigation^20^.

Few studies have described the changes in the sperm cell chromosomes topology in men with reproductive failures and the correct somatic karyotype^21^,^22^,^23^,^24^,^25^,^26^. The observed disorders in spermatozoa from males with idiopathic infertility have been ascribed to (1) atypical packaging of chromosomal territories, (2) instability of chromosomes topology and (3) a lack of interaction between the telomeres. In studies carried out in sperm cells obtained from patients with oligo-astheno-teratozospermia, a change in the intranuclear topology of the X and Y chromosomes was observed in half of the cases^24^. Moreover, Perdrix *et al.*^26^ demonstrated that large vacuoles in spermatozoa are associated with changes in the centromeric area. In immotile sperm cells, the 3D-image analysis showed an altered position of the chromosome 17 centromere and high nuclear/chromocentre volumes^22^. However, some authors have noted that the observed topological differences between infertile and fertile males are rather modest and that a defined pattern of nuclear reorganization of the centromeric loci appeared to be a robust, despite the fact that spermatogenesis is severely compromised in these cases^27^.

Few data found in the literature indicate the influence of chromosome abnormalities on the intranuclear architecture of human spermatozoa. In our earlier studies, we showed the presence of dislocated centromere positions in the studied chromosomes in human spermatozoa with aneuploidies or small additional marker chromosome in infertile patients^25^,^28^. Moreover, we found changes in the spatial arrangement of chromosomes with structural aberrations in spermatozoa obtained from reciprocal translocation carriers^29^. In the spermatozoa of translocation carriers, the chromocentre area was enlarger than in controll sperm cells^29^.

Several studies have shown that the ‘poor’ sperm DNA quality appears to be an important factor affecting male reproductive ability^30^,^31^,^32^,^33^,^34^. Moreover, a meta-analysis has shown that a high-degree sperm of DNA fragmentation has a detrimental effect on IVF/ICSI outcome: high sperm DNA damage was associated with low pregnancy rates in IVF but not in ICSI cycles but was associated with high miscarriage rates in ref. 35 Sperm DNA fragmentation can be attributed to various pathological conditions including: local and systemic diseases, environmental factors, sperm preparation protocols and infection/inflammation in the male reproductive tract^36^,^37^. Sperm DNA integrity can be disrupted by at least three mechanisms: defective chromatin packaging, apoptosis and oxidative stress^33^,^36^.

The aim of this study was to determine whether the deficient spermatogenesis that is manifested by ‘poor’ sperm DNA/chromatin quality may interfere with chromosome topology changes in sperm cells. To detect sperm DNA/chromatin damage we used TUNEL and SCSA assays. To visualize the centromeres of chosen sperm chromosomes we used the FISH method with directly labelled probes. We analyzed the radial localization of thecentromeres of chromosomes 7, 9, 15, 18, X and Y in the chromocenter area of sperm nuclei obtained from five infertile males exhibiting a high degree of sperm DNA fragmentation and from eight control volunteers exhibiting a low degree of sperm DNA fragmentation. We observed changes in the chromocenter area that might have been linked to DNA damages. This conclusion was mainly based on comparisons of the FISH results obtained using TUNEL-positive and TUNEL-negative sperm cells that originated from the same ejaculated sample and that were analyzed on these same slides after the *in situ* reaction of the TUNEL assay.

## Results

### SCSA and TUNEL assays in the patient and control groups

Numerous cut offs associated with the level of DNA damage have been reported in the literature. Currently no absolute limits have been established because the level of DNA fragmentation has not been in unambiguous manner correlated with fertilization and can be similar between cases in which pregnancy occurred and cases in which the women did not conceive or miscarry. Currently, most authors accept the threshold value to be a DFI value of 25% using an SCSA assay^30^,^38^ and 5–10% of sperm cells exhibiting DNA damage as observed using a TUNEL assay^39^.

A group of infertile patients (64males) was subjected to DNA fragmentation analysis using SCSA and TUNEL assays. For this group, the mean value P of DFI was 14.0% (SD ± 13.2%), and HDS was 5.83% (SD ± 4.5%) (Table 1). The mean total frequency of sperm cells with DNA fragmentation indicated by TUNEL assay was 29.3% (SD ± 2.3%). Twenty percent of the 64 males exhibited negative results (25% for SCSA and 10% for TUNEL assays), and only 10% exhibited highly negative results (arbitrarily assumed to be 40% for SCSA and 30% for TUNEL assays) was only 10% (individual data not shown).

Out of this group, four patients (coded P1–P4) with a high rate of DNA fragmentation (Table 2) and whose partners had two unsuccessful IVF procedures were chosen; and at a later stage of the study, the chromosomal topology in the spermatozoa of these patients was analysed. Table 2 shows individual SCSA and TUNEL assay results for patients P1–P4.

A group of 30 fertile males serving as controls were subjected to DNA fragmentation analyses using SCSA and TUNEL assays (Table 1, C values for n = 30). The results obtained for the control group are as follows (Table 1): the mean C value of DFI was 5.4% (SD ± 2.3%), and the mean C value of HDS was 2.7% (SD ± 22.3%). The mean total frequency of sperm cells exhibiting DNA fragmentation as indicated by the TUNEL assay results was 7.45% (SD ± 4.07%). Individual results obtained using the SCSA and TUNEL assays are shown in Table 2 only for control males coded C1–C7 because the study of the sperm cells chromosomal topology was carried out in this group of males. For the six volunteers (C1–C6, Table 2) the results of the SCSA and TUNEL tests did not differ from the mean control values C (Table 1). However, control male C7 exhibited significantly higher values of DFI and TUNEL assay results, the value of DFI was 34.6% and the frequency of sperm cells exhibiting DNA fragmentation as indicated by the TUNEL assay was 9.4%. Both assays were repeated over several months with similar results. Control male C7 presented normal ejaculate parameters^40^ and was the father of three young children. C7 was interesting to the extent that it fitted into the literature discussion regarding the threshold values above which sperm DNA damage can be assumed to be significant for reproduction.

### Radial centromere positioning within the sperm nuclei of the infertile patients with sperm DNA damage and within the sperm nuclei of the control males

**Figure 1:**
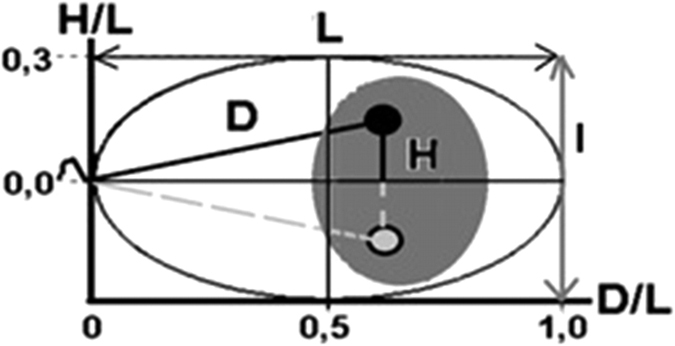
A scheme of the sperm cell nucleus illustrating the specified radial (spatial) localization of the centromeres by measuring of 2D parameters according to the literature^15^. The scheme includes the followinggeometric parameters of sperm cell: L–the length of the long axis; l–the length of the lateral axis; (the L/l ratio describes the shape of the nucleus); D–the distance between the centromere and the attachment site of the tail; H–the distance from the oblong axis. The black point (•) and the light point (o) indicate a centromere of a given chromosome on both sides of the oblong axis (the mirror image). The grey background illustrates the potential area of the location of the different chromosome centromeres on both sides of the longitudinal axis (mirror image) in a singleton sperm cell, which together form the chromocentre. The D/L and H/L ratios can be interpreted as follows: D/L = 1.0 indicates a location near the apical end of a sperm cell; D/L = 0.0 indicates a location close to the tail; H/L = 0.5 indicates the most peripherally localization and H/L = 0.0 indicates a central localization within the sperm nucleus^15^.

The radial localizations of the centromeres of chromosomes 7, 9, 15, 18, X and Y was examined for four selected patients P1–P4 exhibiting a high degree of damaged sperm DNA. The parameters necessary for examining of the radial localization were obtained according to the scheme presented in Fig. 1 (i.e. according to the method described in Zalenskaya & Zalensky^15^. In each case (P1–P4) and for each chromosomes, at least 50 sperm nuclei were measured. The mean values of 50 measurements for individuals P1–P4 and the mean values (P for n = 4) of measurements for the examined group are presented in Table 3.

**Figure 2:**
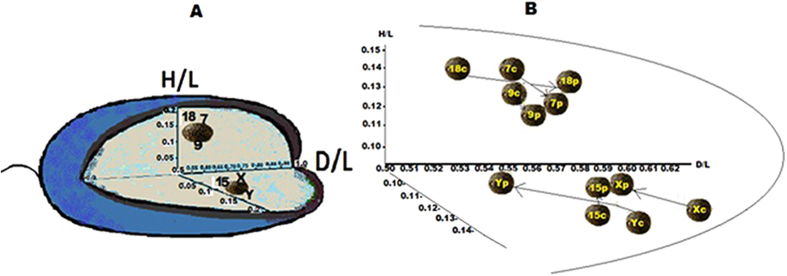
(**A**) Illustration of the preferential radial topology of the centromeres of the chromosomes 7, 9, 18, 15, X and Y within the sperm nuclei of the control fertile males. The circled dark spots illustrate two fragments of the chromocentre area, in which the studied centromeres were localized (without considering the mirror reflection at the side of the oblong axis). The geometric parameters that were used to describe the intranuclear localization of the centromeres are described in Fig. 1; the normalized coordinates H/L and D/L mean C values for control group (i.e. the preferential values) are from Table 3. For ease of comparison, the illustration shows the centromeres of chromosomes X and Y in the same nucleus, however, it should be noted that in the normal sperm nucleus only X or Y is present. (**B**) Comparison of the radial localization of centromeres of chromosomes 7, 9, 15, 18, X and Y between thein control group (spots labelled C) and in the group of selected infertile patients (spots labelled P). Spots show the mean values of C and P which are presented in Table 3; considerable differences between spots C and P are marked with arrows.

**Figure 3:**
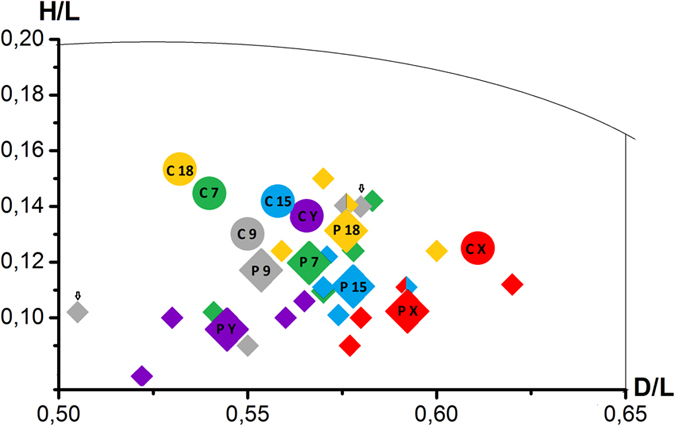
Schematic visualization of the radial localization of the centromeres of chromosomes 7, 9, 15, 18, X and Y based on the basis of all values shown in Table 3. Round spots labelled C illustrate the mean control C values (i.e. preferential values for the examined chromosomes), diamonds labelled P illustrate the mean values of P, and unlabeled small diamonds illustrate individual values for selected infertile patients. Each spot represents the experimentally determined normalized coordinates D/L and H/L as explained in Fig. 1. All mean of P values are significantly different from mean of C values (p ≤ 0.01) (see Table 3) and individual spots that differ significantly from the results from mean P value are marked with arrows.

The radial localizations of the centromeres of chromosomes 7, 8, 9, 15, 18, X and Y were also examined for seven control fertile males C1–C7. The sperm nuclei parameters were measured in the same way as as described for patients P1–P4, according to the scheme given in Fig. 1. Similarly, in each case and for each chromosome, at least 50 sperm nuclei were measured. The results of the preferential radial localization (the mean C value (n = 7)) are presented in Table 3 (for individual values of radial localization within sperm nuclei for the control men C1–C7, see Supplementary Table S1 and Supplementary Fig. S1). Illustrations of the preferential radial topology based on the mean C values presented in Table 3 are extrapolated in Fig. 2A. On the basis of these results, the centromeres topology of the control and fertile sperm nuclei were compared. That is, the mean P values and individual values from P1–P4 are compared to mean control C values in Table 3 (as illustrated in Fig. 2B, and a schematic representation of the centromere radial localization created on the basis of all values shown in Table 3 is presented in Fig. 3). For the H/L parameters, statistically significant differences were found between mean the mean P and C values for the centromeres of all analyzed chromosomes (7, 9, 15, 18, X, and Y (Table 3). For the D/L parameters, no differences were found between the mean P and C values for the centromeres of only chromosomes 9 and X. Additionally, considerable interindividual differences were observed between the D/L and/or H/L parameters. For instance, in patient P1, the H/L values for chromosomes 7, X and Y were significantly different from those of most of the other patients. Interindividual differences between P1–P4 were not found only for H/L for chromosome 15 and for D/L for chromosome 18 (Table 3).

Considering that the intranuclear radial localization of centromeres is determined by two parameters,namely D/L and H/L, we observed that the localization of the examined centromeres was altered in patients P1–P4 when compared to the preferential mean C values (Table 3).

### Preferential radial centromere positioning within the sperm nuclei with and without DNA damage

**Figure 4:**
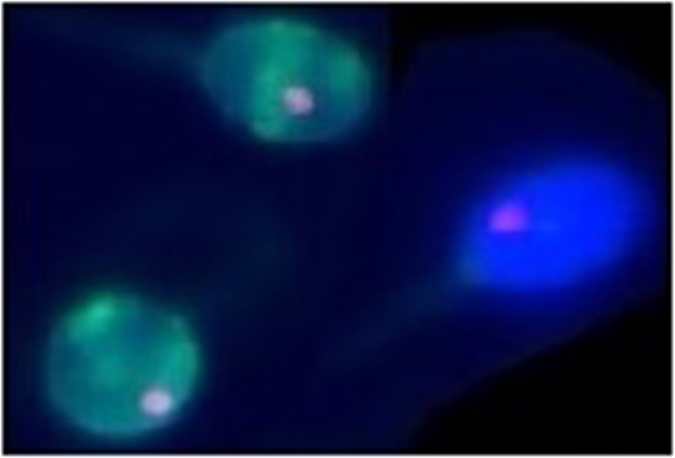
Examples of centromere FISH signals for chromosome 7 (probe TexasRed/DAPI) in two subpopulations of sperm nuclei: TUNEL–positive (T+) (bright green cells) and TUNEL–negative (T−) (blue cell).

Additional experiments were performed to determine whether the observed differences in the centromeres topology of the examined chromosomes in the group of patients P1–P4 were associated with a higher DNA fragmentation of the sperm chromosome. In the first stage, the TUNEL reaction was carried out on sperm cells that were stabilized on microscopic slides. Under the UV light of the fluorescence microscope, only the sperm cells with damaged DNA, (TUNEL–positive (T+) sperm cells), emitted the green lightr (FITC); TUNEL-negative (T−) sperm cells emitted blue light(DAPI). The reaction was sufficiently stable to perform a FISH reaction using centromeric probes (Fig. 4).

**Figure 5:**
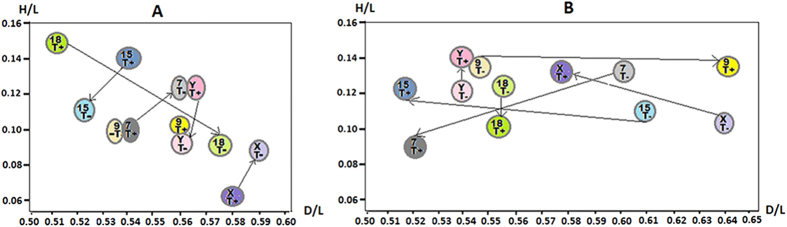
Comparison of the radial localization of the centromeres of chromosomes 7, 9, 15, 18, X and Y within sperm nuclei with and without high DNA fragmentation level. Spots T(+) (TUNEL-positive) and T(−) (TUNEL-negative) represent normalized coordinates for H/L and D/L as explained in Fig. 1, for the values shown in Table 4. Considerable differences between T(+) and T(−) spots are marked with arrows. For ease of comparison, the illustration shows the centromere of chromosomes X and Y in at the same nucleus however, it should be noted that in the normal sperm nucleus only X or Y is present. (**A**) An illustration based on the values for T(+) and (T−) presented in Table 4 for the control fertile male C7 with high DNA fragmentation. (**B**) An illustration based on the values for T(+) and (T−) presented in Table 4 for the patient P2.

This enabled us to distinguish TUNEL-positive (T+) sperm cells (exhibiting a considerable degree of DNA fragmentation) from TUNEL-negative (T−) (normal) sperm cells and to separately measure the localization of the centromeres. Such analyses were carried out in spermatozoa obtained from the control male C7 (Table 2) and the patient P2 (Table 2). We examined the topology of chromosomes 7, 9, 15, 18, X and Y centromeres. The results of the radial localization are presented in Table 4. In the case of the localization in sperm cells from both the control male C7 and the patient P2, significant differences between (T+) and (T−) sperm cells were observed (Table 4). The detected differences involved most of the results obtained; however, slightly fewer differences were observed for the control male C7: no simultaneous differences for D/L and H/L values were observed only for the centromere of the chromosome 9 of C7; differences both for D/L and H/L values were found only for the centromere of chromosome 18 (Table 4). Differences for H/L values only were found for the centromeres of chromosomes 7, 15, X and Y (Table 4). In patient P2, the differences were found for D/L and H/L in the centromeres of chromosomes 7 and X. Differences for D/L values only were found for the centromeres of chromosomes 9 and 15 and differenced for H/L values were found for the centromeres of chromosomes 18 and X (Table 3). The radial localization created on the basis of the T(+) and T(−) values for control C7 and patient P2 (Table 4) are illustrated in Fig. 5.

## Discussion.

In this study, we tested the hypothesis that the topology of centromeres in the chromocentre is altered in the sperm cells of men exhibiting deficient spermatogenesis as manifested by ‘poor’ sperm DNA/chromatin quality. In the sperm nuclei of four infertile patients, we showed that a topological repositioning of the examined centromeres within the chromocentre was associated with DNA damage (Fig. 2B). The conclusion that the observed changes in the chromocentre might be associated with a DNA damage was confirmed by the differences in the centromeres topology between sperm nuclei with or without a DNA fragmentation in sperm cells. Importantly, sperm nuclei originating from the same ejaculated sample were analyzed using FISH following the TUNEL reaction using the same microscopic slides (Fig. 5A,B).

Our observations suggest that a normal DNA fragmentation level is important for the formation and/or maintenance of normal/correct chromosome topology in sperm nuclei. The intranuclear architecture of sperm cells nuclei is well-documented, however, themechanisms governing its structure (i.e. organization and ordering) remain unclear^14^. The chromocentre is created during spermiogenesis and it is very possible that this can be just the factor which creates a defined nuclear topology. Starting in round spermatids, the chromocentre is created by the coalescence of the peri-centromeric heterochromatin of each chromatid in the centre of the nucleus, while their telomeres are associated mostly with the nuclear envelope. At the same time both p and q arms of chromosomes are not randomly organized around the chromocenter, but most often the whole chromosomal domains are arranged in parallel to the chromocentre^14^,^16^,^41^. The chromocentre remains intact throughout spermiogenesis and it is present at the centre of the mature sperm nucleus^42^. The mechanisms that establish and maintaining the chromocentre during spermiogenesis are unknown. According to the commonly used classification, 6 types of spermatids occur: Sa, Sb1, Sb2, Sc, Sd1, and Sd2^43^. Chromatin condensation occurs from Sb1; from the Sb2 stage, visible changes in nuclear shape occur, and the nucleus significantly changes shape in Sc. It can be assumed that the topology of chromosomes in elongated spermatids (Sd1–Sd2) are already shaped similarly to those of mature sperm cells. It thereforeappears that these DNA alterations which might affect the topology of centromeres, must act on chromatin earlier than the elongated spermatids Sd2 stage. It has been shown that multiple chromocentres are a prominent feature of the aberrant spermatid nuclei^44^. In addition, in immotile spermatozoa chromocenter formation is disrupted^21^. Whether multiple chromocenteres are the result of a centromeric heterochromatin spreading or the result of a failure of the centromeres to coalesce into one area remains unknown.

The repositioning of centromeres of selected chromosomes in sperm nuclei with ‘poor’ DNA/chromatin quality most likely mirrors the changes in the chromocentre that occurred earlier than at the Sd2 stage of the spermiogenesis. However, because the reconstruction of the somatic chromatin into sperm chromatin, which causes physiological DNA fragmentation, occurs at the same stage of spermiogenesis as the formation of the chromocentre area, it is not possible to clearly determine the cause and effect relationship between centromere repositioning and sperm DNA damage.

It is important to remember that the aetiology of sperm DNA damage is multifactorial and that different mechanisms may contribute in various proportions to an individual DNA fragmentation index in a given ejaculate. Regardless of the environmental factors (e.g., tobacco smoking) DNA breaks can occur during spermatogenesis but are be normally repaired in most cells^31^,^45^. Theoretically, the checkpoint systems within the shroud of the Sertoli cell cytoplasm should ensure that only spermatozoa with intact chromatin would undergo spermiation. When the checkpoint system fails, the epididymis should scavenge the resulting compromised cells. Three hypotheses have been proposed to explain the presence of a relatively high number of spermatozoa with fragmented DNA in the ejaculate^6^,^46^,^47^. According to the first hypothesis ejaculated spermatozoa with a DNA fragmentation might result from the failure of spermatozoa to mature normally^48^. The replacement of histones by protamines during spermiogenesis involves the formation of endogenous DNA nicks and double-stranded breaks in elongating spermatids and it is necessary for sperm chromatin condensation and a high genome compaction. The second hypothesis relates a DNA fragmentation to the impact of excessive amounts of reactive oxygen species (ROS). In mature spermatozoa, sperm mitochondria represent a major source of a major source of reactive oxygen species (ROS), however, the mitochondrial and cytoplasmic space become physically separated of from the sperm nucleus^49^. According to the third hypothesis, a DNA fragmentation is a consequence of “abortive apoptosis”, referring to the process by which some differentiating germ cells, instead of undergoing complete apoptosis, undergo a restricted form of this process leading to a DNA fragmentation; in this process, the cells can differentiate into mature functional spermatozoa that may retain the capacity for fertilization. Furthermore, the presence of these apoptotic spermatozoa (and also factors associated with infection/inflammation) in the male genital tract may be also an additional source of excessive amount of ROS that can damage DNA^48^,^50^,^51^.

Currently available assays for measuring DNA damage can only determine the amount of a DNA fragmentation that takes place, but none of these assays can differentiate physiological DNA nicks from clinically important and/or pathological nicks. We applied the flow cytometric SCSA and TUNEL assays and also an *in situ* TUNEL assay to detect DNA fragmentation in ejaculated spermatozoa. We used the modified TUNEL assay as proposed by Mitchell *el al*.^52^. which better defines the DNA damage status. Based on the donut model for sperm chromatin structure, Sharman and Ward^53^ predicted that the most of the DNA breaks identified by the SCSA assay are located in the toroid linker region and suggested that the SCSA assay would have a similar ability to detect ss and ds DNA breaks as the TUNEL assay. The latter assay is limited to studying areas of sperm chromatin that are accessible to enzyme modifications. Because these areas are probably the most active sites after fertilization when sperm chromatin is decondensed, the results of a TUNEL assay can be closely correlated with human infertility^53^.

Numerous reports over the past 10–15 years have shown that a ‘poor’ sperm DNA/chromatin quality is higher in infertile males than in fertile males and that thisinferior quality is associated both with low natural and/or ART pregnancy rates^30^,^32^,^34^,^54^. Our study also found (Table 1) that the rate of DNA fragmentation was higher in ejaculated sperm cells among the 64 infertile patients than among the 30 control males thus confirming the previously reported results. Although a small percentage of spermatozoa from fertile males also possess detectable levels of a DNA fragmentation, the case of control male C7 (Table 2) (with undoubted paternity), who exhibited a similarly high level of DNA damage as the infertile patients, drew our attention. Therefore, the control male C7 was selected for further careful study (Table 4, Fig. 5A).

The findings presented here indicated that an increased level of a DNA damage might be implicated in sperm altered chromosome topology with potentially adverse effect upon the sperm function. At the same time, it is not yet possible to unambiguously determine the effect of the discovered centromere localization changes (Figs 2B and 5) on the sperm cell function. An additional difficulty is that the centromere repositioning of was associated with a ‘poor’ sperm DNA quality; it is therefore possible that a cause and effect relationship exist between these abnormalities. It is possible to assume that the sperm cells from patients P1–P4 (Table 2) retained the ability to penetrate the oocytes during the *in vitro* fertilization (IVF), although the created embryos/fetuses were not developing. In turn, the ability of the sperm cells to penetrate an oocytes (*in vivo*) was undoubted for donor C7, who was the father of three children. Some previously reported data have indicated that in some sperm exhibiting radiation-induced DNA damage, fertilization was not blocked^55^. These studies are consistent with the finding that sperm DNA damage has minimal effects on pronuclear formation and syngamy^56^. Because spermatozoa have no known DNA repair mechanisms it is clear that the integrity of the paternal genome depends on the capacity of the oocyte to recognize DNA damage and the potential to repair it. Indeed, some breaks in the sperm DNA can be repaired by the oocyte; however, if the level of a sperm DNA damage exceeds the oocyte’s capacity to repair it, then the cells might undergo apoptosis^57^. In compromised oocytes the capacity to repair the paternal DNA may not be available. It has been shown that the paternal effect of a DNA damage is more prominent in the maternal aged group with potentially suboptimal oocytes^56^. Due to the observation that the effect of a paternal DNA damage is more evident during the later stages of embryogenesis, one could suggest that in patients P1–P4 the combination of damaged sperm DNA and oocytes with suboptimal DNA repair (a possible scenario for oocytes after induced ovulation) was the only reason preventing embryo development. However, an intriguing influence of sperm DNA damage was also observed during the first stages of embryonic development when the embryonic genome was not yet activated^56^. Moreover, some data indicate that paternal DNA damage might favour the induction of aberrations in the paternal chromosomes after the first metaphase^58^,^59^. It is very likely that during this period (when the embryonic genome is nor activated) the indicated repositioning of paternal centromeres can interfere with the normal cell division / development of the early embryo. Thus, it can be suggested that in addition to sperm DNA damage and the oocyte’s insufficient competence of the oocyte to repair all “paternal mistakes”, a third factor (incorrect topology of the sperm chromosomes) may be involved in reproductive failures.

In conclusion, the relationship between the organization and function of the sperm chromatin is at an early stage of discovery. It is also important to remember that the analysis of chromosomes in ejaculated spermatozoa only provides information about those anomalies that escaped elimination during meiosis. For this reason, studies of ejaculated sperm cells investigate the effects of rather than the mechanisms that lead to the observed disorders.

The data described here show that a high level of sperm DNA fragmentation might be associated with the observed changes in the chromocentre, which were most probably were formed before the Sd2 stage of spermiogenesis. Because we analysed the topology of only 5 out of the 23 sperm chromosome centromeres, this study provides an incomplete picture of all the possible reshuffling variations. However, reshuffling phenomenon undoubtedly occurs in chromocenter in the presence of DNA breaks. It is well known that the aetiology of sperm DNA damage is multi-factorial. Some of the mechanisms that alter sperm DNA can operate in spermatozoa with already shaped nuclear architecture; but such alterations cannot be distinguished by the assays currently used. In addition, it cannot be excluded that chromosome repositioning might also generate some damage in the tightly compact chromatin that characterizes the mature sperm nucleus, however, this notion remains speculative.

## Materials and Methods

### Male participants

This study was approved by the Local Bioethical Committee of the Medical University of Poznań, Poland, and informed consent was obtained from all subjects. All the procedures performed within our project were conducted in the accordance with the approved by Committee protocols. The collected male population, for which sperm DNA quality tests (SCSA and TUNEL assays) were performed, comprised of 64 infertile and of 30 fertile men (Table 1). The group of infertile men were aged between 30 to 40 years with normal karyotypes; the men had suffered at least two failed *in vitro* fertilization (IVF) procedures. As controls, semen samples were obtained from 30 healthy normozoospermic males between 23 to 30 years of age with proven fertility and normal karyotype. The topology analyses of the selected chromosome centromeres were performed in sperm nuclei of four infertile patients (coded P1–P4 in Table 2) with normozoospermia and in seven control volunteers (coded C1–C7 in Table 2) from males with known of DNA quality test results (Table 1).

### Semen collection and processing

Semen samples were obtained by masturbation after 3–5 days of sexual abstinence. Within 60 minutes after ejaculation and liquefaction, the routine semen analyses were performed according to the World Health Organization 2010 criteria^40^. The peroxidative test was used for the leukocytes assessment in the ejaculate^60^. Spermatozoa from collected semen samples were separated from seminal plasma by centrifugation at 600 g for 8 minutes. An aliquot of sperm suspensions was washed twice in TNE buffer (10 mM Tris-HCl, 150 mM NaCl, 1mM EDTA, pH 7.5) and processed for use in the sperm chromatin structure assay (SCSA). The remaining part of the sperm suspensions was washed twice in phosphate buffer saline (PBS) pH 7.4 (Biomed, Lublin, Poland) and processed for use in the TUNEL-assay.

### Sperm chromatin structure assay (SCSA)

After washing in TNE buffer, sperm samples were treated with acid-detergent solution (0.08 N HCl, 150 mM NaCl, 0.1% Triton-X 100, pH 1.2) to induce DNA denaturation. After exactly 30 s, acridine orange (AO, Sigma, St. Louis, USA) staining solution (0.6 mg AO, 0.1M citric acid, 0.2M Na_2_PO_4_, 1mM EDTA, 0.15 M NaCl, pH 6.0) was added to sperm suspension^30^. Flow cytometry analysis was performed using a Beckman Coulter flow cytometer (Cell LabQuanta SC MPL, Beckman Coulter, Fullerton, CA, USA) equipped with a 488-nm argon-ion laser. For each analysis, at least 20,000 events were collected, at a rate of 200–250 events/sec. were collected and analysed using Cell LabQuanta SC MPL Analysis software (Beckman Coulter). The percentage of sperm cells with DNA fragmentation emitting strong red fluorescence (% DFI) and the percentage of incompletely condensed sperm cells with high DNA staining emitting strong green fluorescence (% HDS) were calculated. Fluorescence measurements were repeated three times using separate samples.

### TUNEL assay

Sperm DNA fragmentation was evaluated using the TUNEL (Terminal deoxynucleotidyl Transferase Biotin-dUTP Nick End Labeling) assay (FlowTACS Apoptosis Detection Kit, Trevigen, Inc., Gaithersburg, MD, USA) following the manufacturer’s instruction as previously described^37^. Sperm samples were fixed using 3.7% formaldehyde and permeabilized using Cytonin. Next, biotinylated nucleotides were added to the free 3′-ends of the DNA fragments in the presence of terminal deoxynucleotidyl transferase (TdT). After a complex had formed between biotinylated DNA fragments and streptavidin-conjugated fluorescein (FITC), the spermatozoa were washed in PBS and analysed using a Beckman Coulter flow cytometer (Cell LabQuanta SC MPL, Beckman Coulter, Fullerton, CA, USA). A minimum of 10,000 events were acquired for each evaluated sample. The percentage of TUNEL-positive cells was determined. To optimize the labelling conditions, TACS-nuclease-pretreated spermatozoa were used as a positive control. An unlabelled experimental negative control sample (sperm exposed to the reaction mixture in the absence of TdT) was used to indicate the level of background fluorescence. The fluorescence measurement was repeated two times using separate samples.

### TUNEL assay–*in situ*

For the patient coded P2 (Table 2) and the control fertile male coded C7 (Table 2) with high levels of sperm DNA damage (as observed using the SCSA and TUNEL assays), TUNEL labelling was performed *in situ*. After washing three times in PBS, the spermatozoa were fixed with a fresh fixative solution (methanol:acetic acid, 3:1 v/v, −20 °C). On the day of the experiment, the fixed sperm samples were spread onto slides and air-dried. After washing twice in PBS, TUNEL was performed using a TACS 2 TdT-Fluor *In Situ* Apoptosis Detection Kit (Trevigen, Inc., Gaithersburg, MD, USA) according to manufacturer’s instructions. Briefly, sperm smears were permeabilized using Cytonin. Next, the sperm smears were incubated in labeling reaction mix containing biotinylated nucleotides, terminal deoxynucleotidyl transferase (TdT) and Mn^2+^. After a complex had formed between the biotinylated DNA fragments and streptavidin-conjugated fluorescein (FITC), the sperm smears were washed twice in PBS and stained with DAPI. To optimize the labelling conditions, a TACS-nuclease-treated positive control was used. An unlabelled experimental control sample was used to indicate the level of background fluorescence associated with non-specific binding of the streptavidin-conjugated FITC.

### FISH (fluorescence *in situ* hybridization) procedure and DNA probes

After liquefaction, aliquots of spermatozoa from the collected fresh semen samples after liquefaction were washed three times in PBS and fixed using cold methanol: acetic acid (3:1 v/v, −20 °C) for 20 min. After two rinses with fresh fixative, thesperm samples were spread onto slides and air-dried. The slides were then washed two times in PBS and plunged into a solution of 10 mM dithiothreitol (DTT) in a 0.1 M Tris-HCL (pH 8.5) for 5–10 min at 43 °C. After this mild procedure the sperm cells exhibited a well-defined boundary and in most cells, the sperm tails were attached to their heads. The slides were rinsed in 2 × SSC, then dehydrated in an ethanol series from 70% to 100% and air-dried.

FISH experiments were performed using red or green directly labelled probes, which were obtained from Cytocell Technologies Ltd. (Cambridge, UK) according to the manufacturer’s protocol. To identify chromosomes 7, 9, 15, 18, X and Y, we used alpha-satellite centromeric probes. The hybridization mixture (3 μl of each probe plus hybridization buffer) was applied to each slide. The probes were covered with a coverslip and sealed with rubber cement. The probes and cellular DNA were then denatured simultaneously for 2 min at 75 °C. Hybridization was carried out overnight in a moist chamber at 37 °C. After hybridization, the slides were washed for 2 min in the solution of 0.4x SCC at 72 °C and then for 30 sec in the 2x SCC/0.5% Tween 20 solution. Counterstaining was performed using DAPI/antifade reagent.

Moreover, FISH experiments in association with *in situ* TUNEL labeling were performed for patient P2 (Table 2) and the control fertile male C7 (Table 2), both with high levels of sperm DNA damage (as observed earlier in the SCSA and TUNEL assays). That is, the FISH labelling reaction was performed on the same microscopic slides immediately after *in situ* TUNEL reaction, thus enabling us to distinguish the centromeric probes from TUNEL-positive and TUNEL-negative spermatozoa in each consecutively analysed cell nucleus.

Hybridization signals were observed using an Olympus Bx41 microscope equipped with a filters for DAPI/FITC/TEXAS Red and an oil-immersed objective (100x, 1.25 NA). The efficiency of FISH was 98%.

### Determination of the radial centromere positioning in sperm nuclei obtained from infertile patients and control volunteers

To determine the preferential radial position of FISH-labelled centromeres of the studied chromosomes, we applied the previously established Zalenskaya and Zalensky’s^15^ graphic model (Fig. 1).

The following geometrical parameters were measured within the nucleus of individual sperm cells: L–length from the tail attachment point to theacrosome (long axis); l–the length of the lateral axis; (the L/l ratio describes the shape of the nucleus); D–the distance from the FISH centromere signal to thetail attachment point; and H–the distance from the FISH centromere signal. D/L and H/L ratios were calculated. The highest D/L values (close to 1.0) indicated that the centromeres were located near an acrosome area. The lowest H/L values (close to 0.0) indicated that the central position of the centromeres were within the sperm nucleus; the higher values indicated more peripheral positions. D/L and H/L ratios described radial intranuclear position of the centromeres of the examined chromosomes^15^. A minimum of 50 informative sperm nuclei (for each single probe in a single FISH assay) were analysed per sample using strict selection criteria: only spermatozoa exhibiting a tail, mild decondensation and having an elliptical head shape (according to the scheme in Fig. 1: L/l ≤ 1.4) were included in the analysis.

### Statistical analysis

The data were analysed using the Statistica,version 7.0 (StatSoft, Tulsa, OK, USA). Non-parametric analysis of variances (Kruskal–Wallis test) was used followed by the Dunnett and Dunn multiple comparisons tests; the threshold value indicating sperm DNA damage was 10% for the TUNEL assay^39^ and 25% of DNA fragmentation index for the SCSA assay^38^. The Mann–Whitney U test, one-way ANOVA, χ^2^ and Fisher tests were used in order to assess the statistical significance of the results describing the radial distribution of spermatozoan centromeres with and without DNA damage and to compare the data obtained for the control group of volunteers and the infertile patients. Differences were considered statistically significant at *P*-value ≤ 0.01.

## Additional Information

**How to cite this article**: Wiland, E. *et al.* Topology of chromosome centromeres in human sperm nuclei with high levels of DNA damage. *Sci. Rep.* **6**, 31614; doi: 10.1038/srep31614 (2016).

## Supplementary Material

Supplementary Information

## Figures and Tables

**Table 1:** Mean values of the SCSA and TUNEL assays results for sperm cells from n = 64 infertile patients and n = 30 fertile controls.

	SCSA		TUNEL
	DFI (%)	HDS (%)	(%)
Infertile patients:			
Mean value P	14.0*	5.83	29.3*
(for n = 64) ± SD	±13.2	±4.5	±2.3
Control group:			
Mean control value C	5.4	2.7	7.45
(for n = 30) ± SD	±2.3	±1.7	±4.07

SCSA: Sperm chromatin structure assay; DFI: DNA fragmentation index; HDS: sperm with high DNA stainability; TUNEL: Terminal deoxynucleotidyl Transferase Biotin-dUTP Nick End Labeling (sum of cells with DNA fragmentation); SD: standard dilution. Value p ≤ 0.01 was considered to be statistically significant.

^*^Values significantly higher from mean control value C (p < 0.01).

**Table 2:** Individual results of SCSA and TUNEL assay results for sperm cells from infertile patients P1–P4 exhibiting high degree of DNA fragmentation and from fertile control volunteers C1–C7 for whom the studies of sperm chromosomes topology were performed.

	SCSA		TUNEL
	DFI (%)	HDS (%)	(%)
Patients P1–P4			
P1	41.4*	13.5*	45.1*
P2	54.6*	7.2*	25.0*
P3	33.7*	17.4*	26.3*
P4	38.7*	1.4	37.4*
Control volunteers C1–C7			
C1	8.0	2.4	5.5
C2	2.3	1.7	2.9
C3	3.2	2.0	5.5
C4	6.2	3.3	2.2
C5	4.0	2.9	6.5
C6	4.3	1.2	1.1
C7	34.6**	2.1	50.0**

SCSA: Sperm chromatin structure assay; DFI: DNA fragmentation index; HDS: sperm with high DNA stainability; TUNEL: Terminal deoxynucleotidyl Transferase Biotin-dUTP Nick End Labeling (sum of cells with DNA fragmentation); Value p ≤ 0.01 was considered to be statistically significant.

^*^Values significantly higher from mean control value C (p < 0.01).

^**^Values C7 were significantly higher than the remaining results in the control group and mean value P (p < 0.001).

**Table 3:** Comparison of the preferential radial localization of the centromeres of chromosomes 7, 9, 15, 18, X and Y (and also individual values) within the sperm nuclei of infertile patients exhibiting high DNA fragmentation (P1–P4 in Table 2) with preferential localization in control fertile men (mean value for C1–C7 in Table 2; (individual values for C1–C7 are shown in Supplementary Table S1). The parameters D/L and H/L are as described in Fig. 1.

Chromosome	7		9		15		18		X		Y	
Patients	D/L	H/L	D/L	H/L	D/L	H/L	D/L	H/L	D/L	H/L	D/L	H/L
**P1**	**0.541**^**1**^	**0.102**^**2,C**^	**0.550**	**0.090**^**4,C**^	**0.592**^**C**^	**0.111**^**C**^	**0.600**^**C**^	**0.124**^**6,C**^	**0.577**^**7,C**^	**0.090**^**8,C**^	**0.522**^**9,C**^	**0.079**^**10,C**^
**P2**	**0.570**^**C**^	**0.110**	**0.576**	**0.141**^**5**^	**0.570**	**0.111**^**C**^	**0.559**	**0.124**^**6,C**^	**0.620**	**0.112**	**0.565**	**0.106**^**C**^
**P3**	**0.578**^**C**^	**0.124**	**0.505**^**3,P,C**^	**0.102**^**C**^	**0.574**	**0.101**^**C**^	**0.570**^**C**^	**0.150**	**0.580**^**7,C**^	**0.100**^**C**^	**0.530**^**C**^	**0.100**^**C**^
**P4**	**0.583**^**C**^	**0.142**	**0.580**^**P,C**^	**0.140**^**5**^	**0.571**	**0.122**	**0.576**^**C**^	**0.140**	**0.592**	**0.111**	**0.560**	**0.100**^**C**^
**Mean value P** ± *SE* (P1–P4 in Tab. 2)	0.567*±*0.007*	0.120*±*0.005*	**0.553**±*0.007*	0.118*±*0.005*	0.577*±*0.006*	0.111*±*0.003*	0.576*±*0.006*	0.135*±*0.003*	**0.592**±*0.008*	0.103*±*0.004*	0.544*±*0.007*	0.096*±*0.004*
**Mean value C** ± *SE* (C1–C7 in Tab. 2)	**0.540**±*0.006*	**0.145**^**D**^±*0.005*	**0.550**±*0.005*	**0.130**±*0.003*	**0.560**±*0.007*	**0.140**^**D**^±*0.003*	**0.532**^**C**^±*0.006*	**0.153**^**E**^±*0.003*	**0.611**^**A**^±*0.007*	**0.125**±*0.004*	**0.566**^**B**^±*0.007*	**0.137**±*0.004*

Individual values **D/L** and **H/L** for FISH signals of centromeres are the average of the measurements taken in at least 50 sperm nuclei for each of the chromosomes in each patient. *SE*–standard error. One-way ANOVA test was used to compare results (p value ≤ 0.01 was considered to be statistically significant). *Mean values P significantly different from mean control value C (p ≤ 0.01); ^**C**^values significantly different from mean control value C (p ≤ 0.01); ^**P**^values significantly different from mean control value P(p ≤ 0.01); ^**1**^Significantly different from P3, and P4 results (p = 0.003 and p = 0.01); ^**2**^Significantly different from P3, and P4 results (p = 0.003; p = 0.000 and p = 0.000); ^**3**^Significantly different from P1, P2, and P4 results (p = 0.01; p = 0.000; p = 0.000 and P = 0.000); ^**4**^Significantly different from P2 and P4 results (p = 0.000); ^**5**^Significantly different from P3 results (p = 0.001); ^**6**^Significantly different from P3 result (p = 0.01); ^**7**^Significantly different from P2 result (p = 0.01); ^**8**^Significantly different from P2 and P4 results (p = 0.01); ^**9**^Significantly different from P4 result (p = 0.005); ^**10**^Significantly different from P2, P3 and P4 results (p = 0.005; p = 0.007 and p = 0.007); ^**A**^Mean D/L value for chromosome X significantly different from D/L results for chromosome 7, 9, 15, 18 and Y (p = 0.000); ^**B**^Mean D/L value for chromosome Y significantly different from D/L results for chromosome 7, 18 and X (p = 0.001; and p = 0.000); ^**C**^Mean D/L value for chromosome 18 significantly different from D/L results for chromosome 15, X and Y (p = 0.002; and p = 0.000); ^**D**^Mean H/L value for chromosomes 7 and 15 significantly different from H/L results for chromosome 9, 18, X and Y (p = 0.000; p = 0.01, p = 0.000 and p = 0.01); ^**E**^Mean H/L value for 18 chromosome significantly different from H/L results for chromosome 9; X and Y (p = 0.000).

**Table 4:** Comparison of the preferential radial localization of the centromeres of chromosomes 7, 9, 15, 18, X and Y within the sperm nuclei exhibiting or not exhibiting high DNA fragmentation level (TUNEL-positive and TUNEL-negative spermatozoa, respectively).

Chromosome	7		9		15		18		X		Y	
	D/L	H/L	D/L	H/L	D/L	H/L	D/L	H/L	D/L	H/L	D/L	H/L
	mean		mean		mean		mean		mean		mean	
Value **C7 T(+)** ± *SE* **TUNEL (+) positive = **spermatozoa with high DNA fragmentation level	**0.540**±*0.010*	**0.100**^1^*±*0.007*	**0.561**±*0.011*	**0.102**±*0.008*	**0.541**±*0.015*	**0.140**^**2**^±*0.008*	**0.512**±*0.016*	**0.149**^4^*±*0.006*	0.580*±*0.015*	**0.060**^5^*±*0.007*	**0.562**±*0.013*	**0.122**±*0.009*
Value **C7 T**(−) ± *SE* **TUNEL** (−) **negative = **spermatozoa with no DNA fragmentation	**0.562**±*0.010*	**0.123**±*0.005*	**0.533**±*0.010*	**0.100**±*0.005*	**0.521**±*0.014*	**0.114**±*0.006*	**0.577**^3^*±*0.016*	**0.093**±*0.004*	**0.591**±*0.015*	**0.090**±*0.004*	^**0.560**^±*0.013*	**0.090**^**6**^±*0.008*
Value **C7** ± *SE* spermatozoa not fractionated	**0.571**±*0.010*	**0.135**±*0.002*	**0.566**±*0.009*	**0.105**±*0.005*	**0.554**±*0.014*	**0.109**±*0.005*	**0.500**±*0.015*	**0.118**±*0.003*	**0.626**±*0.014*	**0.102**±*0.006*	**0.573**±*0.012*	**0.123**±*0.008*
Value **P2 T(+)** ± *SE* **TUNEL (+) positive = **spermatozoa with high DNA fragmentation level	**0.521**^7^*±*0.010*	**0.090**^8^*±*0.005*	**0.645**^9^*±*0.007*	**0.134**±*0.005*	**0.514**^10^*±*0.012*	**0.122**±*0.005*	**0.552***0.010*±	**0.105**^11^*±*0.005*	**0.580**^12^*±*0.011*	**0.130**^13^*±*0.005*	**0.539**±*0.013*	**0.139**^14^*±0.006
Value **P2 T**(−) ± *SE* **TUNEL** (−) **negative = **spermatozoa with no DNA fragmentation	**0.602**±*0.007*	0.137*±*0.005*	**0.546**±*0.012*	**0.136**±*0.004*	0.610*±*0.008*	**0.110**±*0.004*	**0.557**±*0.008*	**0.125**±*0.005*	**0.640**±*0.007*	**0.100**±*0.004*	**0.538**±*0.010*	**0.123**±*0.006*
Value **P2** ± SE spermatozoa not fractionated	**0.570**±*0.007*	**0.110**±*0.005*	**0.576**±*0.007*	**0.141**±*0.004*	**0.570**±*0.008*	**0.111**±*0.004*	**0.559**±*0.008*	**0.124**±*0.005*	**0.620**±*0.007*	**0.112**±*0.005*	**0.565**±*0.009*	**0.106**±*0.006*

The spermatozoa originated from the control C7 and from the infertile patient P2 (Results of the TUNEL assays for C7 and P2 are presented in Table 2). The parameters D/L and H/L are as described in Fig. 1. Individual values **D/L** and **H/L** for FISH signals of centromeres are the average of the measurements taken in at least 50 sperm nuclei for each of the chromosomes in case of each analyzed male. *SE*–standard error. One-way ANOVA test was used to compare results (p ≤ 0.01 was considered to be statistically significant). *Value significantly different from value for spermatozoa not fractionated respectively from C8 or P2 (p ≤ 0.01); ^**1**^Significantly different from C7 T(−) and C7 result (p = 0.005 and p = 0.000); ^**2**^Significantly different from C7 T(−) and C7 results (p = 0.001 and p = 0.000); ^**3**^Significantly different from C7 T(+) and C7 results (p = 0.000); ^**4**^Significantly different from C7 T(−) and C7 results (p = 0.000); ^**5**^Significantly different from C7 T(−) and C7 results (p = 0.000); ^**6**^Significantly different from C7 T(+) and C7 results (p = 0.000); ^**7**^Significantly different from P2 T(−) and P2 results (p = 0.000 and p = 0.002); ^**8**^Significantly different from P2 T(−) and P2 results (p = 0.000 and p = 0.01); ^**9**^Significantly different from P2 T(−) (p = 0.000); ^**10**^Significantly different from P2 T(−) and P2 results (p = 0.000); ^**11**^Significantly different from P2 T(−) and P2 results (p = 0.01 and p = 0.007); ^**12**^Significantly different from P2 T(−) and P2 results (p = 0.000 and p = 0.01); ^**13**^Significantly different from P2 T(−) and P2 results (p = 0.000 and p = 0.001); ^**14**^Significantly different from P2 T(−) and P2 results (p = 0.009 and p = 0.000).
