# Supplementary material for: Topology of chromosome centromeres in human sperm nuclei with high levels of DNA damage
Source: Sci Rep. 2016 Aug 25;6:31614. doi: 10.1038/srep31614 (PMC4997348; doi:10.1038/srep31614)
Supplement: Supplementary Information [file srep31614-s1.doc]

**Supplementary files for manuscript:**

**Topology of chromosome centromeres in human sperm nuclei with high levels of DNA damage.**

**Ewa Wiland, Monika Fraczek, Marta Olszewska & Maciej Kurpisz ***

Institute of Human Genetics, Polish Academy of Sciences, Department of Reproductive Biology and Stem Cells, Strzeszyńska 32, 60-479 Poznań, Poland

*Correspondence and requests for materials should be addressed to M.K.: (e-mail: [kurpimac@man.poznan.pl](mailto:kurpimac@man.poznan.pl), and to the first author: [ewila@man.poznan.pl](mailto:ewila@man.poznan.pl)).

**Supplementary Table S1** Individual values of the radial localization of the centromeres of chromosomes 7, 9, 15, 18, X and Y within sperm nuclei from control fertile men**)**.The parameters D/L and H/L are as described in **Fig. 1**.

| **Chromosome** | | **7** | | | **9** | | **15** | | **18** | | **X** | | **Y** | |
| --- | --- | --- | --- | --- | --- | --- | --- | --- | --- | --- | --- | --- | --- | --- |
| **Control volunteers** |  | | **D/L** | **H/L** | **D/L** | **H/L** | **D/L** | **H/L** | **D/L** | **H/L** | **D/L** | **H/L** | **D/L** | **H/L** |
| **C1** |  | | **0.533** | **0.157** | **0.550** | **0.134** | **0.511*1** | **0.173*2** | **0.543** | **0.173** | **0.634** | **0.140** | **0.577** | **0.157** |
| **C2** |  | | **0.553** | **0.146** | **0.562** | **0.124** | **0.550** | **0.139** | **0.573*4** | **0.163** | **0.609** | **0.133** | **0.627*9** | **0.148** |
| **C3** |  | | **0.531** | **0.138** | **0.525** | **0.125** | **0.600** | **0.144** | **0.510** | **0.169** | **0.627** | **0.100** | **0.513*10** | **0.126** |
| **C4** |  | | **0.536** | **0.153** | **0.552** | **0.153** | **0.562** | **0.155** | **0.500** | **0.157** | **0.607** | **0.140** | **0.522*11** | **0.158** |
| **C5** |  | | **0.535** | **0.150** | **0.540** | **0.130** | **0.580** | **0.128** | **0.580*5** | **0.139** | **0.620** | **0.100** | **0.604** | **0.104*12** |
| **C6** |  | | **0.526** | **0.134** | **0.560** | **0.144** | **0.554** | **0.129** | **0.520** | **0.149** | **0.560*7** | **0.157*8** | **0.564** | **0.144** |
| **C7** |  | | **0.571** | **0.135** | **0.566** | **0.105** | **0.554** | **0.109*3** | **0.500** | **0.118*6** | **0.626** | **0.102** | **0.573** | **0.123** |
| **Mean value C** *±SE*  (C1-C7 in Tab. 2) |  | | **0.540**  *±0.006* | **0.145D**  *±0.005* | **0.550**  *±0.005* | **0.130**  *±0.003* | **0.560**  *±0.007* | **0.140D**  *±0.003* | **0.532C**  *±0.006* | **0.153E**  *±0.003* | **0.611A**  *±0.007* | **0.125**  *±0.004* | **0.566B**  *±0.007* | **0.137**  *±0.004* |

Individual values **D/L** and **H/L** for FISH signals of centromeres are the average of the measurements taken in at least 50 sperm nuclei for each of the chromosomes in each control man. *SE*–standard error. One-way ANOVA test was used to compare results (p value≤0.01 was considered to be statistically significant).

*Individual D/L or H/L values significantly different from mean control value C (p≤ 0.01);

*1p=0.003; *2p=0.010; *3p=0.010; *****4 p=0.010; *5 p=0.003; *****6 p=0.004; *****7 p=0.002; *****8 p=0.009; *****9 p=0.000; *10 p=0.001; *11 p=0.007; *12­p=0.007.

**A** Mean D/L value for chromosome X significantly different from D/L results for chromosome 7, 9, 15, 18 and Y (p=0.000);

**B** Mean D/L value for chromosome Y significantly different from D/L results for chromosome 7, 18 and X (p=0.001; and p=0.000);

**C** Mean D/L value for chromosome 18 significantly different from D/L results for chromosome 15, X and Y (p=0.002; and p=0.000);

**D** Mean H/L value for chromosomes 7 and 15 significantly different from H/L results for chromosome 9, 18, X and Y (p=0.000; p=0.01, p=0.000 and p=0.01);

**E** Mean H/L value for 18 chromosome significantly different from H/L results for chromosome 9; X and Y (p=0.000);


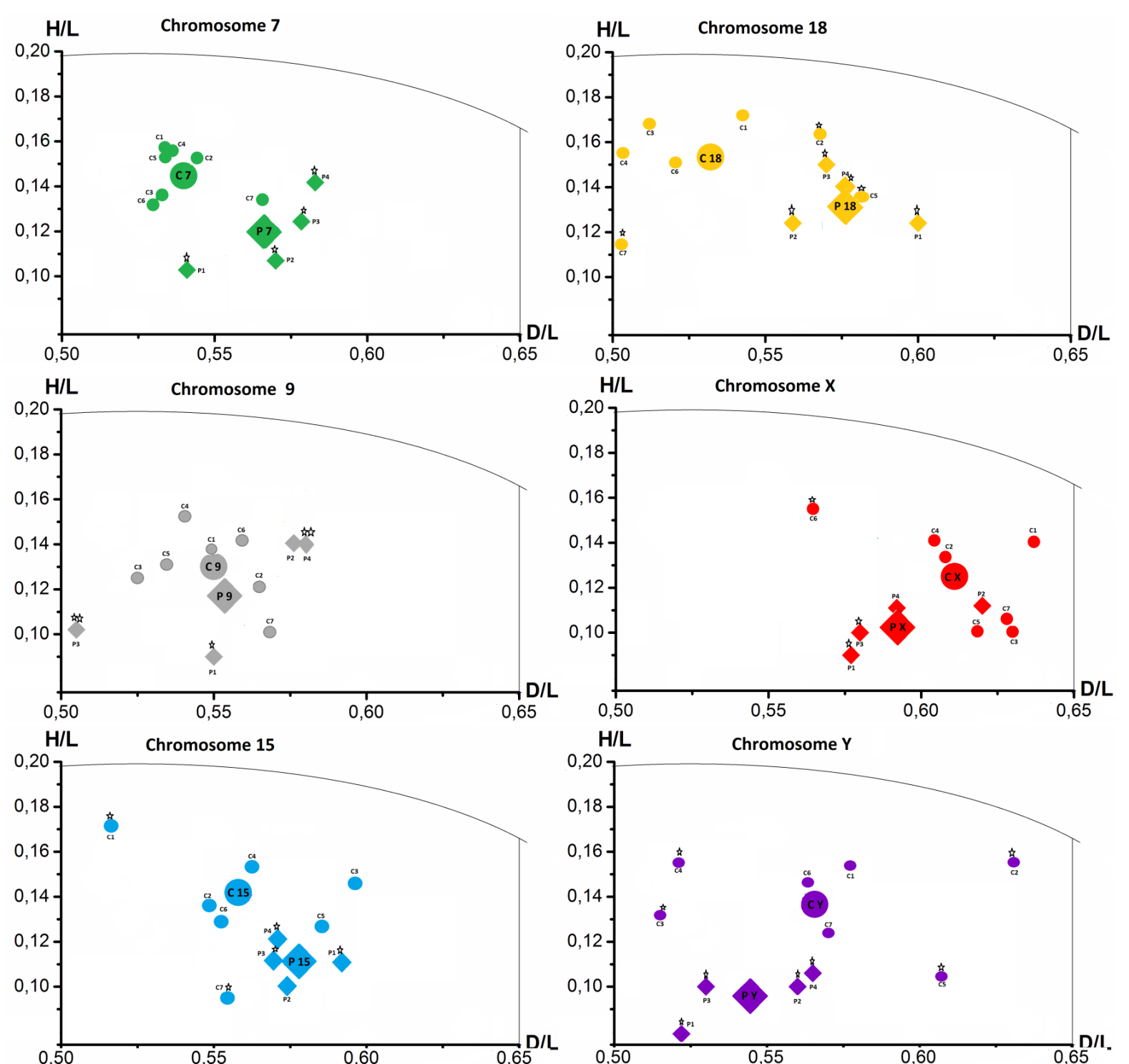
**Supplementary Figure S1** Comparison of the radial localization of the centromeres of chromosomes 7, 9, 15, 18, X and Y within the conrol sperm nuclei (round spots) (C1 –C7, mean C values) with that of infertile patients (diamonds) (P1 –P4, mean P values). The geometric parameters that have been used to describe the intranuclear localization of the centromeres are as described in **Fig. 1**. The coordinates H/L and D/L of the patients (individual P1 –P4 and mean P values) are presented in **Table 3,** and those for the control group (individual C1 –C7 and mean C values) are presented in **Supplementary Table S1**. Individual spots P1 –P4 and also C1 – C7 that differ significantly from the mean preferential value C for a given chromosome, are marked with an asterisk. For chromosome 9 individual P3 and P4 values that differ significantly from the mean values of both C and P are marked with a double asterisk. For each chromosome, the mean value of P is significantly different from the mean value of C (p≤ 0.01) (see **Table 3**).
